# Year-round supplementation of direct-fed microbials to cow-calf pairs and its effects on maternal and offspring productive performance

**DOI:** 10.1093/tas/txaf137

**Published:** 2025-10-04

**Authors:** Philipe Moriel, Vinicius S Izquierdo, Cassio C Brauner, João L Silva, Luciana M Sousa, Júlio G Berwanger, Kauani B Cardoso, Conner A Crawford, João H J Bittar, Pedro Monteiro, Matthew H Poore, Reinaldo F Cooke, João M B Vendramini, Bruno I Cappellozza

**Affiliations:** University of Florida/IFAS, Range Cattle Research and Education Center, Ona, FL, 33865, United States; University of Florida/IFAS, Range Cattle Research and Education Center, Ona, FL, 33865, United States; University of Florida/IFAS, Range Cattle Research and Education Center, Ona, FL, 33865, United States; University of Florida/IFAS, Range Cattle Research and Education Center, Ona, FL, 33865, United States; University of Florida/IFAS, Range Cattle Research and Education Center, Ona, FL, 33865, United States; University of Florida/IFAS, Range Cattle Research and Education Center, Ona, FL, 33865, United States; University of Florida/IFAS, Range Cattle Research and Education Center, Ona, FL, 33865, United States; University of Florida/IFAS, Range Cattle Research and Education Center, Ona, FL, 33865, United States; College of Veterinary Medicine, University of Florida, Gainesville, FL, 32610, United States; College of Veterinary Medicine, University of Florida, Gainesville, FL, 32610, United States; Department of Animal Science, North Carolina State University, Raleigh, NC, 27695, United States; Department of Animal Science, Texas A&M University, College Station, TX, 77843, United States; Texas A&M AgriLife Research & Extension Center, Stephenville, TX, 76401, United States; Novonesis, Lyngby, 2800, Denmark

**Keywords:** *Bacillus*, *Bos indicus*, cow-calf, post-weaning, preweaning

## Abstract

On day 0 (94 ± 19 days prepartum), 296 Brangus beef cows (7 ± 3 years of age; <25% *Bos indicus*) were stratified by body weight (**BW**; 537 ± 56 kg) and body condition score (**BCS**; 5.46 ± 0.74) and randomly assigned to 1 of 26 bahiagrass pastures (11 to 12 cows and 8.1 to 9.6 ha per pasture). On day 0, treatments were randomly assigned to pastures (13 pastures per treatment) and consisted of free-choice access to a trace mineral supplement, either alone (**CON**) or combined (**BAC**) with 3 g per head daily of a *Bacillus*-based DFM supplement (Bovacillus; Novonesis, Lyngby, Denmark) from day 0 to 330 (weaning). At weaning, 48 steers and 64 heifers were selected (3 steers and 4 heifers per pasture; 16 pastures) for the post-weaning phase. Steers were assigned to preconditioning (day 350 to 398) and feedlot periods (day 399 to 609), whereas heifers were developed from day 350 until pregnancy diagnosis (day 615). Non-binary and binary data were analyzed using the MIXED and GLIMMIX procedure of SAS, respectively. supplement DM intake was greater (*P *≤ 0.03) for BAC vs. CON cow-calf pairs on days 42 to 48, 56 to 62, 168 to 174, and 266 to 272. Cow BCS at the end of the breeding season was greater (*P *= 0.01) for BAC vs. CON cows, whereas all cow reproductive data and prepartum plasma data did not differ (*P *≥ 0.13) between treatments. Serum immunoglobulin G concentrations at birth, preweaning average daily gain (**ADG**), and BW at weaning were greater (*P *≤ 0.05) for BAC vs. CON calves. Steer growth performance during preconditioning did not differ (*P *≥ 0.18) between treatments, but serum titers against infectious bovine rhinotracheitis and parainfluenza 3 viruses were greater (*P *= 0.05) for BAC vs. CON steers 19 days after first vaccination. Steer feedlot performance and carcass traits did not differ between treatments (*P *≥ 0.16), except for carcasses grading Low Choice or above, which were greater (*P *= 0.05) for BAC vs. CON steers. Despite the greater (*P *= 0.03) pre-breeding ADG of CON vs. BAC heifers, the percentage of mature BW at breeding, puberty status, and pregnancy percentages did not differ (*P *≥ 0.17) between treatments. Thus, year-round supplementation of *Bacillus*-based DFM via a mineral delivery method improved cow BCS during breeding and enhanced calf preweaning growth. Although no reproductive benefits were observed for cows and their heifers, DFM supplementation increased post-weaning immune response and carcass quality of steers.

## Teaser

Feeding *Bacillus*-based probiotics year-round through mineral supplements improved cow body condition and calf growth before weaning. These early-life benefits also enhanced steer immune response and carcass quality without affecting reproduction in cows and their heifers.

## Introduction

Direct-fed microbials (**DFM**) have emerged as a practical supplementation strategy to improve ruminal fermentation, promote the establishment of beneficial microbial populations, and enhance fiber and overall nutrient digestibility (Pan et al 2022; Cappellozza et al 2023a, 2023b; Limede et al., 2025). Within this category, *Bacillus* spp. have attracted particular interest because they not only influence rumen function but also provide benefits throughout the gastrointestinal tract. These benefits include inhibiting pathogen growth, stimulating mucin and biofilm formation (Santano et al 2020; Segura et al 2020; Capern et al 2025), and producing digestive enzymes and anti-inflammatory compounds that can strengthen the intestinal barrier and maintain gut integrity (Rhayat et al 2019; Luise et al 2022). Moreover, early-life gut microbial colonization represents a critical period during which these benefits could have lasting effects. Although the mammalian gastrointestinal tract is sterile in utero, colonization begins rapidly at birth and shapes the development of the gastrointestinal and immune systems, with consequences for long-term growth, health, and productivity (Rodriguez et al 2015; Magalhães et al 2024; Cappellozza et al 2025). This process of gut colonization is influenced by both maternal and offspring diet, suggesting that DFM supplementation in cow–calf systems has the potential to benefit both generations.

A common limitation of many DFM products is their inability to maintain viability beyond 24 hours after exposure to environmental conditions. The *Bacillus*-based DFM used in the present study has been shown to remain viable under challenging conditions. Cappellozza et al. (2023a) demonstrated their stability when exposed to high temperatures during pelletization and when stored for up to 12 months in a mineral–vitamin premix containing vitamins, macro, and trace minerals. This technology allows for consistent delivery of viable bacterial cells under practical field conditions.

Despite the potential of *Bacillus*-based DFM, few studies have evaluated the broader impacts of this strategy on productive performance of cow–calf pairs. Maternal supplementation during late gestation and early lactation in nulliparous beef cows has been shown to improve maternal body condition score (**BCS**) before calving and to enhance calf post-weaning growth and vaccine-induced immune response (Izquierdo et al 2024, 2025). However, these earlier studies implemented a shorter supplementation period, limited to late gestation and early lactation, and did not provide calves with preweaning access to DFM supplements. Building on this foundation, we hypothesized that extending *Bacillus*-based DFM supplementation to a year-round protocol and delivering it via trace mineral supplementation accessible to both cows and calves would amplify the benefits to maternal and offspring performance previously reported by Izquierdo et al. (2024, 2025). Our objectives were to evaluate the effects of year-round supplementation of *Bacillus*-based DFM, delivered through a free-choice mineral supplement, on: (1) BCS and reproduction of multiparous beef cows; (2) preweaning growth of their calves; and (3) post-weaning growth, immune response, and carcass traits of steers, and growth and reproduction of heifers.

## Materials and methods

The experiment was conducted at the University of Florida, Institute of Food and Agricultural Sciences, Range Cattle Research and Education Center, Ona, Florida (27°23′N and 81°56′W) and at the North Carolina State University (Butner Beef Cattle Field Laboratory; 36°10ʹN and 78°48ʹW) from August 2023 to April 2025. All animals used in this experiment were cared for by practices approved by the University of Florida—Institute of Animal Care and Use Committee (protocol #202300000563).

### Animals and diets

#### Precalving period

On day 0 (94 ± 19 days prepartum), 296 multiparous, fall-calving Brangus crossbred beef cows (7 ± 3 years of age; <25% *Bos indicus*) were enrolled in the study. These cows were previously bred by 16 Brangus bulls (3 to 7 years of age) during a 90-day natural breeding season. Cows were stratified by bull, body weight (**BW**; 537 ± 56 kg) and BCS (5.46 ± 0.74; scale of 1 to 9), and randomly assigned to two blocks: Block 1 = 176 cows allocated to 16 bahiagrass (*Paspalum notatum)* pastures (11 cows and 8.1 ha per pasture); and Block 2 = 120 cows allocated to 10 bahiagrass pastures (12 cows and 9.6 ha per pasture). Different stocking rates were used to account for the initial herbage mass of each block, despite the similar previous grazing management and fertilization. On day 0, treatments were randomly assigned to pastures within each block (8 and 5 pastures per treatment in blocks 1 and 2, respectively). Treatments consisted of free choice access to a trace mineral supplement, either alone (**CON**) or combined (**BAC**) with a DFM supplement containing a mix of *Bacillus subtilis* 810 and *B. licheniformis* 809 from day 0 to 330 (calf weaning). The trace mineral supplement (expected intake of 51 g per head daily) met the requirements of beef cows (NASEM 2016) and included: 17% Ca, 4% P, 21% NaCl, 1% Mg, 60 mg/kg Co, 1750 mg/kg Cu, 350 mg/kg I, 60 mg/kg Se, 5000 mg/kg Zn, 441 IU/g Vitamin A, 33 IU/g Vitamin D_3_, and 0.44 IU/g of Vitamin E; (Vigortone, Brookville, OH). The commercial DFM supplement (Bovacillus; Novonesis, Lyngby, Denmark) was blended once weekly with the trace mineral supplement at equivalent amounts to ensure a daily intake of 3 g of DFM per head, based on the expected 51 g of the trace mineral supplement. This BAC formulation was designed to provide an expected total colony forming unit (**CFU**) of 6.6 × 10^9^ per head daily (Izquierdo et al 2024, 2025; Terré et al 2024). Supplements were hand-delivered weekly (Mondays at 0800 h) into weather-resistant plastic mineral feeders with self-closing rubber lids (PolyGuard Mineral Feeder, Behlen country, Columbus, NE). Mineral feeders were monitored twice weekly (Wednesdays and Fridays) for signs of rain exposure or hardening. When replacement was necessary, the dry matter (**DM**) concentration of the removed material was determined to estimate supplement DM intake up to the time of removal, after which the respective fresh product was added to ensure continuous free-choice access.

#### Preweaning period

Cows were monitored daily for calving from day 60 to 153. Calves were processed (weighed, tagged, and castrated if male) within 24 hours after birth, but only after consuming the maternal colostrum. Following birth, calves received the same free-choice supplement assigned to their dams on day 0, with an expected intake of 51 g of trace mineral salt per head daily (corresponding to 3 g of DFM per head for BAC calves). supplement delivery continued weekly (Mondays at 0800 h) to allow free-choice consumption from calving until weaning. From day 120 to 240, cow-calf pairs and bulls had free-choice access to limpograss (*Hemarthria altissima*) hay (3.1% crude protein; 53% total digestible nutrients; DM basis) and were limit-fed a sugarcane molasses (*Saccharum officinarum*) and urea supplement (12.4 kg of DM per cow and bull weekly; 82% DM, 22% crude protein and 75% total digestible nutrients; Westway Feed Products LLC, Clewiston, FL). The molasses-urea supplement was formulated to minimize postpartum BCS loss of cows (NASEM 2016; Moriel et al 2024). The weekly amount of molasses-urea supplement was split into two equal portions and delivered every Monday and Thursday at 0800 h in plastic tanks.

Fertile Brangus bulls (5 ± 2 years of age; confirmed via breeding soundness exam 90 days before the start of the breeding season) were placed with cows from day 150 to 240 and rotated among pastures every 30 days. Cow-calf pairs remained in their respective previous prepartum pasture assignment throughout the breeding season. No injuries or mounting difficulty were observed. Cows and calves received an oral drench of fenbendazole (5 mg per kg of BW; Safe-guard, Merck Animal Health, Summit, NJ) on days 240 and 280. Calves were monitored daily by trained personal, and no health problems were detected. All calves were weaned abruptly on day 330 (on average 236 ± 19 days of age).

At weaning, 48 steers and 64 heifers were randomly selected within sex from block 1 (3 steers and 4 heifers per pasture; 16 pastures) for a post-weaning evaluation period. All remaining calves were sold at weaning. The selected steers and heifers were sorted by sex and managed separately on 5- and 10-ha bahiagrass pastures, respectively. From day 330 to 349, steers and heifers were provided concentrate supplementation at 0.5%, 1.0%, and 1.5% of BW (DM basis) for 7, 7, and 6 days, respectively. The concentrate contained 22.25% soybean hulls, 22% soybean meal, 22% dried distillers’ grains, 15% cottonseed hulls pellets, 15% whole corn, 2% Ca carbonate, and 1.75% sugarcane molasses (United Feed Company, Okeechobee, FL, USA). During this adaptation period, 2 CON and 2 BAC steers were removed from the study due to leg injury or sickness not related to the study.

#### Steer preconditioning and feedlot periods

Steers (*n *= 44) were assigned to preconditioning from day 350 to 398 and then a feedlot period from day 399 to 609. On day 350, steers were randomly allocated into 1 of 16 partially covered drylot pens (15 × 5 m; 2 to 3 steers per pen), maintaining their previous pasture group. During preconditioning, steers had free-choice access to limpograss hay and were supplemented with concentrate at 1.5% of BW (DM basis). The concentrate contained 22.25% soybean hulls, 22% soybean meal, 22% dried distillers’ grains, 15% cottonseed hulls pellets, 15% whole corn, 2% Ca carbonate, and 1.75% sugarcane molasses (United Feed Company, Okeechobee, FL, USA). The average nutrient composition of the hay and concentrate is shown in Table 1. During the preconditioning, all steers were provided free-choice access to the CON trace mineral supplement (expected daily intake of 51 g per head). On day 350, steers were treated with doramectin for internal and external parasites (5 mL subcutaneous; Dectomax injectable, Zoetis Inc., Kalamazoo, MI) and vaccinated for bovine respiratory disease (2 mL subcutaneous; Bovi Shield One Shot, Zoetis Inc.) and clostridium (5 mL subcutaneous; Ultrabac 7, Zoetis Inc.). On day 369, steers received subcutaneous booster vaccinations of Bovi Shield Gold 5 (2 mL) and Ultrabac 7 (5 mL; Zoetis Inc.).

**Table 1.** Nutritional composition of concentrate and hay offered to steers from weaning to the end of the preconditioning period (day 330 to 398), total mixed rations (**TMR**) offered to steers during the growing (day 407 to 482) and finishing periods (day 483 to 609), and concentrate offered to heifers during the post-weaning period (day 330 to 615).

Item^a^	Steer preconditioning concentrate	Steer preconditioning hay	Steer growing TMR	Steer finishing TMR	Heifer post-weaning concentrate
**Crude protein, %**	19.8 ± 0.25	5.3 ± 0.51	12.2 ± 0.14	13.3 ± 0.07	20.7 ± 0.36
**Soluble protein, % of crude protein**	13.3 ± 1.53	30.7 ± 2.08	48.0 ± 1.41	49.0 ± 5.66	15.0 ± 1.73
**Acid detergent fiber, %**	32.8 ± 0.80	42.3 ± 0.36	17.0 ± 2.55	8.15 ± 0.64	32.0 ± 0.64
**Neutral detergent fiber, %**	49.5 ± 0.91	71.6 ± 0.55	27.1 ± 3.54	14.7 ± 1.06	47.4 ± 0.44
**Total digestible nutrients, %**	72.0 ± 0.12	54.0 ± 0.20	73.0 ± 1.41	77.5 ± 0.71	72.0 ± 0.10
**NEm, Mcal/kg DM**	1.66 ± 0.01	0.98 ± 0.01	1.77 ± 0.04	1.91 ± 0.01	1.69 ± 0.01
**NEg, Mcal/kg DM**	1.05 ± 0.01	0.44 ± 0.01	1.15 ± 0.04	1.27 ± 0.01	1.08 ± 0.01
**Ca, %**	1.39 ± 0.10	0.42 ± 0.06	0.66 ± 0.12	0.48 ± 0.08	1.46 ± 0.07
**P, %**	0.46 ± 0.02	0.19 ± 0.01	0.34 ± 0.02	0.31 ± 0.04	0.43 ± 0.01
**Mg, %**	0.29 ± 0.01	0.20 ± 0.02	0.17 ± 0.02	0.12 ± 0.01	0.27 ± 0.01
**K, %**	1.54 ± 0.02	0.62 ± 0.05	1.18 ± 0.12	0.60 ± 0.11	1.51 ± 0.02
**Na, %**	0.16 ± 0.01	0.07 ± 0.01	0.12 ± 0.01	0.14 ± 0.01	0.13 ± 0.01
**S, %**	0.38 ± 0.01	0.16 ± 0.02	0.14 ± 0.01	0.13 ± 0.01	0.36 ± 0.01
**Fe, mg/kg**	317 ± 7.51	119 ± 19.6	192 ± 18.8	173 ± 74.3	308 ± 6.81
**Zn, mg/kg**	50.0 ± 1.00	23.3 ± 1.15	56.0 ± 8.49	60.0 ± 0.01	54.3 ± 1.15
**Cu, mg/kg**	6.33 ± 0.58	7.67 ± 0.58	25.5 ± 2.12	28.5 ± 3.54	7.00 ± 0.10
**Mn, mg/kg**	20.7 ± 1.15	17.7 ± 2.31	39.5 ± 10.6	30.5 ± 6.36	22.0 ± 0.08
**Mo, mg/kg**	1.57 ± 0.38	1.33 ± 0.40	1.10 ± 0.28	0.85 ± 0.07	2.10 ± 0.10

a Samples of concentrate and hay offered to steers were collected on days 350, 370, and 390, samples of TMR were collected every 14 days within the growing and finishing periods, and samples of concentrate offered to heifers were collected on days 330, 464, and 582. All samples were analyzed at a commercial laboratory (Dairy One Forage Laboratory, Ithaca, NY) for wet chemistry analysis.

On day 398, all steers were supplemented with concentrate at 1.5% of BW at 0900 h and provided free choice access to water and limpograss hay for 8 hours. At 1700 h on day 398, steers were loaded into a commercial livestock trailer and transported for 1193 km over 14 hours to the feedlot facility at North Carolina State University (Butner Beef Cattle Field Laboratory; 36°10ʹN and 78°48ʹW) where they were housed for the remainder of the experiment. Immediately following arrival (0700 h on day 399), steers were weighed and assigned to 1 of 16 covered concrete slatted floor pens (130 m^2^ and 2 to 3 steers per pen) using the same preweaning pasture group. Steers were provided free-choice access to corn silage (*Zea mays*; 33.5% DM; 72% total digestible nutrients and 9.4% crude protein) and water for 8 days. The feedlot period was divided into a growing period from day 407 to 482, followed by a finishing period from day 483 to 609. The growing and finishing total mixed rations (**TMR**; Table 1) were provided once daily at 0800 h. The growing TMR (DM basis) consisted of 78% corn-silage, 9.1% coarse cracked corn, 11% soybean meal, 1.6% limestone, 0.3% trace mineral salt, 22 mg/kg of monensin (Rumensin 90, Elanco Animal Health, Greenfield, IN) and 2200 IU/kg of Vitamin A. Steers remained on the growing TMR for 76 days. Steers were adapted to the finishing TMR over a 7-day period, receiving a 50:50 mix of the growing and finishing TMR (DM basis). The finishing TMR (DM basis) consisted of 15% corn-silage, 79.2% coarse cracked corn, 3% soybean meal, 1% urea, 1.5% limestone, 0.3% trace mineral salt, 33 mg/kg of monensin (Rumensin 90, Elanco Animal Health, Greenfield, IN) and 2200 IU/kg of Vitamin A. Steers remained on the finishing TMR for 126 days. Steers were monitored daily for signs of illness by trained personnel during the entire feedlot period. On day 609, all steers were loaded into a livestock trailer on the same day and transported for 790 km to a commercial packing facility for harvest (Cargill Wyalusing Protein Processing Plant, Wyalusing, PA).

#### Heifer post-weaning period

On day 350, heifers were stratified by their previous pasture group (4 heifers per pasture; 8 pastures per treatment) and randomly assigned to 1 of 16 bahiagrass pastures (1 ha and 4 heifers per pasture). Heifers received concentrate DM supplementation at 1.5% of BW from day 350 until pregnancy diagnosis (day 615). The concentrate contained 22.25% soybean hulls, 22% soybean meal, 22% dried distillers’ grains, 15% cottonseed hulls pellets, 15% whole corn, 2% Ca carbonate, and 1.75% sugarcane molasses (United Feed Company, Okeechobee, FL, USA). The average nutrient composition of the concentrate offered to heifers is shown in Table 1. This supplementation strategy was designed to support an average daily gain (**ADG**) of 0.60 to 0.70 kg/day (Moriel et al 2022). All heifers were provided free-choice access to the CON trace mineral supplement from day 330 to 615 (expected daily intake of 51 g per head). On day 464, heifers were assigned to an estrus synchronization protocol. The protocol consisted of intramuscular injection of 25 mg of prostaglandin F_2α_ (PGF_2α_; 5 mL Lutalyse; Zoetis Animal Health, Florham Park, NJ, USA) on day 464, insertion of an intravaginal controlled internal drug release (**CIDR**; 1.38 g P4; Zoetis) and intramuscular injection of 100 µg of gonadotropin-releasing hormone (**GnRH**; 2 mL Factrel; Zoetis) on day 468, and then CIDR removal and intramuscular injection of 25 mg of PGF_2α_ (5 mL Lutalyse) on day 475. Estrus detection patches (Estrotect; Rockway Inc., Spring Valley, WI, USA) were applied to all heifers on day 475 to detect the estrus activity from days 475 to 478. Heifers were considered to be in estrus when at least 50% of the rub-off coating was removed from the patch or absent. Heifers detected in estrus between days 475 and 478 were inseminated (**AI**) 12 hours after estrus detection using semen from a single Angus sire (Select Sires, Plant City, OH, USA). On day 478, all heifers previously not observed in estrus received an intramuscular injection of 100 µg of GnRH (2 mL Factrel) and were fixed-time AI using semen from a single Angus sire. Yearling Angus bulls were placed with heifers on day 485 (1 bull per pasture). Bulls were rotated among pastures every 28 days until day 582 to remove any potential bull effect. All bulls successfully passed a breeding soundness exam 60 days before the start of the breeding season.

### Data and sample collection

#### Supplemental treatments

The *Bacillus*-based DFM was stored under controlled temperature and humidity to ensure product stability. From day 0 to 330, weekly samples of CON and BAC supplements were collected from each pasture (immediately after supplement delivery to pastures), pooled by treatment, and sent to Novonesis (Milwaukee, WI) for the analysis of *Bacillus* spore concentration using the European standard EN-15784 methodology (2021). This approach ensured that CON supplements contained a minimal presence of *Bacillus* spores (<1.0 × 10^5^ CFU/g) and BAC supplements met the target dose (CFU = 6.6 × 10^9^ per head daily). supplement intake (g per head daily) was calculated weekly from day 0 to 330 by subtracting the weekly refusals from the amount of supplement offered in the previous week and then dividing by 7. Weekly samples of CON and BAC supplements offered and refused were collected from each pasture and dried at 60 °C for 72 hours to determine the DM concentration.

#### Pastures

Herbage mass (Gonzalez et al 1990) and hand-plucked samples of pastures were collected every 30 days from day 0 to 300, excluding the hay feeding period (day 120 to 240). Forage samples were analyzed for concentrations of crude protein and in vitro digestible organic matter. Herbage allowance was calculated as the total herbage mass divided by the combined BW of cows and calves per pasture (Sollenberger et al 2005).

#### Cows

Full BW and BCS were recorded for each cow before morning supplementation on days 0, 55, 150, 240, and 330. Cow BCS was assessed by the same two technicians during the study. Shrunk BW was not obtained to avoid prenatal stress that could interfere with fetal development and calf postnatal performance (Carroll et al 2021). Blood samples (10 mL) were collected from the same 3 cows per pasture via jugular venipuncture into sodium-heparin containing tubes (Vacutainer, Becton Dickinson; Franklin Lakes, NJ) on days 0, 30, and 60 to determine the plasma concentrations of cortisol, glucose, urea N, insulin-like growth factor 1 (**IGF-1**) and non-esterified fatty acids (**NEFA**). The percentage of cows calving their first offspring (calves that were in utero from day 0 to 94) and their second offspring during the study (calves that were conceived from day 150 to 240) was calculated based on cows diagnosed as pregnant and delivering a live calf. Percentage of cows pregnant with their second offspring was assessed on day 280 via rectal palpation by a trained veterinarian. Cows that did not deliver a live first offspring (8 CON and 9 BAC cows) were excluded from all post-calving statistical analyses.

#### Calf preweaning

Full BW was recorded for each calf within 24 hours after birth and on days 150, 240, and 330. At birth, blood samples (10 mL) were collected from 2 steers and 2 heifers per pasture (Block 1 only) via jugular venipuncture into: (1) sodium-heparin containing tubes (Vacutainer, Becton Dickinson) to determine the plasma concentrations of cortisol and haptoglobin; and (2) additive-free tubes (Vacutainer, Becton Dickinson) to determine the serum concentrations of immunoglobulin G (**IgG**).

#### Steer preconditioning

Shrunk BW was recorded for each steer on days 350 and 398 after 12 hours of feed and water withdrawal. Full BW was recorded for each steer on days 369 and 383 to adjust the concentrate DM offered per pen. Hay DMI was calculated daily for each pen by subtracting the hay amount refused from the hay amount offered. Samples of hay offered and refused were collected daily from each pen and dried for 48 hours at 56 °C to determine the DM concentration. Samples of concentrate and hay were collected before morning feeding on days 350, 370, and 390 to assess the nutritional composition. Blood samples (10 mL) were collected from all steers via jugular venipuncture, 3 to 4 hours after concentrate supplementation, into: (1) sodium-heparin containing tubes (Vacutainer, Becton Dickinson) to determine the plasma concentrations of IGF-1, cortisol and haptoglobin on days 350, 352, 355, 358, 369, and 398; and (2) additive-free tubes (Vacutainer, Becton Dickinson) to determine the serum antibody titers against bovine viral diarrhea virus type 1a (**BVDV-1a**) and 2 (**BVDV-2**), infectious bovine rhinotracheitis (**IBR**) and parainfluenza 3 (**PI-3**) viruses on days 350, 369, and 398. All blood samples were placed on ice immediately after collection and centrifuged within 1 hour at 1200 × g for 25 minutes at 4 °C. Plasma and serum were stored at −20 °C until analysis.

#### Steer feedlot

Individual shrunk BW of steers were recorded immediately after unloading and arrival at the Butner Beef Cattle Field Laboratory (day 399), following the 14-hour transportation period. Individual full BW of steers were recorded on 2 consecutive days at the start of the growing period (days 407 and 408), end of the growing period (day 483 and 484), and before loading and transportation to packing facility (day 608 to 609). Shrunk BW was not collected during growing and finishing periods to avoid disturbing feeding behavior and DM intake. Daily intake (as-fed basis) of growing and finishing TMR was determined by weighing the daily amount of each respective diet offered to each pen at 0800 h and subtracting the remaining orts observed for each pen on the subsequent morning. During the growing and finishing periods, samples of each TMR were collected every 14 days to determine the DM percentage and to calculate the daily DMI of each feedlot pen. Hot carcass weights were recorded immediately following slaughter. All carcasses were chilled for 48 hours before determination of longissimus muscle area, 12^th^ rib fat thickness, and the percentage of kidney, pelvic, and heart fat by a trained United States Department of Agriculture grader. Dressing percentage was calculated individually by dividing the hot carcass weight by the BW of steer on day 609.

#### Heifer post-weaning

Shrunk BW was recorded for each heifer on days 350, 464, and 615 after 12 hours of feed and water withdrawal. Full BW was recorded for each heifer every 28 days from days 378 to 582, immediately before every morning concentrate supplementation, to adjust the concentrate DM offered per pasture. Blood samples (10 mL) were collected from all heifers via jugular venipuncture, 3 to 4 hours after concentrate supplementation, into sodium-heparin containing tubes (Vacutainer, Becton Dickinson) to determine the plasma concentrations of glucose and IGF-1 on days 370, 400, 464, 520, and 582. Transrectal ultrasonography was performed by a veterinarian on: (1) days 434 and 464 (start of estrus synchronization) to assess puberty status, based on the presence of a corpus luteum; (2) day 520 to determine the percentage of heifers pregnant to AI; and (3) day 615 to determine the final pregnancy percentage. Heifers were monitored twice daily for calving, and calving date recorded using Julian calendar dates. Reproductive tract scores (1 to 5 scale; 1 = prepubertal; 5 = pubertal; Anderson et al 1991; Day and Anderson 1998) were evaluated on days 434 and 464 by a trained veterinarian to evaluate the reproductive development of each heifer (Moriel et al 2022). Samples of concentrate were collected on days 330, 464, and 582 for nutrient composition analysis.

### Laboratory analyses

Pastures samples were analyzed in duplicates at the University of Florida Forage Evaluation Support Laboratory to determine the concentration of N (Gallaher et al 1975) and in vitro digestible organic matter (Moore and Mott 1974). Samples of concentrate offered to heifers and concentrate and hay offered to steers during the preconditioning period were placed in a forced-air oven and dried for 72 hours at 56 °C. Dried samples of concentrate and hay were ground to pass through 1- and 4-mm stainless steel screens, respectively, using a Wiley mill (Model 4, Thomas-Wiley Laboratory Mill, Thomas Scientific, Swedesboro, NJ, USA). Concentrate and hay samples were submitted to Dairy One Forage Laboratory (Ithaca, NY) for wet chemistry analysis.

Serum concentrations of IgG were assessed using a bovine-specific ELISA kit (E11-118; Bethyl Laboratories, Inc., Montgomery, TX). Plasma concentrations of IGF-1 were assessed using a commercial human ELISA kit (SG100B; R&D Systems, Inc., Minneapolis, MN, USA), previously validated for bovine samples (Moriel et al 2012). Quantitative colorimetric kits were used to determine the plasma concentrations of glucose and urea N (G7521 and B7551, respectively; Pointe Scientific Inc.; Moriel et al 2015), and NEFA (HR Series NEFA-2; Wako Pure Chemical Industries Ltd, Richmond, VA; Palmer et al 2022). Plasma concentrations of haptoglobin were calculated by assessing the haptoglobin-hemoglobin and differences in peroxidase activity (Cooke and Arthington 2013). Plasma concentrations of cortisol were determined using radioimmunoassay (#07221106, MP Biomedicals, Santa Ana, CA; Colombo et al 2019). All samples were analyzed in duplicate, and reanalyzed when necessary, to ensure inter- and intra-assay coefficients of variation were less than 5%.

Antibody titers against BVDV-1a, BVDV-2, IBR and PI-3 viruses were assessed at the Oklahoma Animal Disease Diagnostic Laboratory (Stillwater, OK) using procedures described by Rosenbaum et al. (1970). Serum samples were assessed to determine the highest dilution at which complete protection of cells against each virus was observed. Antibody titers were expressed as log_2_ values, with dilution ranges spanning from 1:4 to 1:1024. Steers were classified as seropositive (assigned a value of 1) if titers were ≥4 (log_2_), and seronegative (assigned a value of 0) if titers were <4. These binary scores were used to calculate the percentage of steers exhibiting positive seroconversion against each virus (Richeson et al 2008; Moriel et al 2016).

### Statistical analyses

Pasture was considered the experimental unit for all statistical analyses. Non-binary and binary data were analyzed as a randomized complete block study using the MIXED and GLIMMIX procedures of SAS (SAS Institute Inc., Cary, NC, USA, version 9.4), respectively. The Satterthwaite approximation was used to determine the denominator degrees of freedom for the test of fixed effects. All results are reported as least-square means. All data were separated using PDIFF if a significant *F*-test was detected. Significance was set at *P *≤ 0.05, and tendencies were noted if *P *> 0.05 and ≤ 0.10.

#### Precalving and preweaning

Random effects included pasture(treatment × block) and cow(pasture) or calf(pasture), except for pasture data, which included only pasture(treatment × block). Repeated measures (e.g., forage data, supplement intake, BW, BCS, and cow plasma data) were tested for fixed effects of block, treatment, day, and all resulting interactions. Autoregressive 1 covariance structure was utilized because it generated the lowest Akaike information criterion. Calf BW and blood data at birth, calf ADG, and cow BCS and BW change were tested for fixed effects of block, treatment, and treatment × block. All calf data was covariate-adjusted for calf sex and age, whereas cow plasma, BW, and BCS data were covariate-adjusted for its respective baseline data obtained on day 0. Binary data (pregnancy and calving percentages, and male calves at birth) were tested for fixed effects of block, treatment, and treatment × block.

#### Steer post-weaning

Random effects included pasture(treatment) and steer(pen). Repeated measures (e.g., steer BW, intake, and blood data) were tested for fixed effects of treatment, day, and treatment × day, using the compound symmetry covariance structure. Steer ADG, gain: feed, and carcass variables were tested for fixed effects of treatment. Steer BW and ADG were covariate-adjusted for steer age.

#### Heifer post-weaning

Random effects included pasture(treatment) and heifer(pasture). Repeated measures (e.g., heifer BW, plasma data, reproductive tract score, and puberty attainment) were tested for fixed effects of treatment, day, and treatment × day, using the compound symmetry covariance structure. Post-weaning ADG and mature BW at breeding were tested for fixed effects of treatment. Heifer BW and ADG were covariate-adjusted for heifer age. Heifers detected in estrus and pregnancy percentages were tested for fixed effects of treatment.

## Results

Effects of block × treatment × day, block × treatment, and block × day were not detected (*P *≥ 0.19) for any variable analyzed in the study.

### Pasture and supplement

**Fig. 1.**
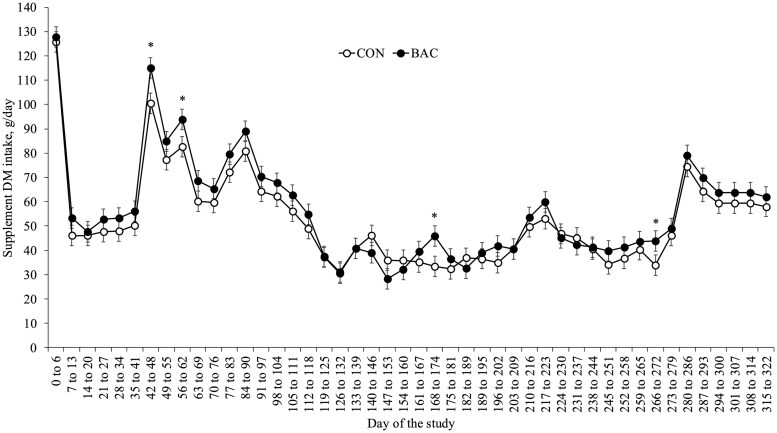
Supplement dry matter (**DM**) intake of cow-calf pairs offered free-choice access to trace mineral supplement, either alone (**CON**) or combined (**BAC**) with a DFM supplement (3 g/head/day) containing a mixture of *Bacillus subtilis* and *B. licheniformis* (6.6 × 10^9^ CFU per day) from day 0 (94 ± 19 days prepartum) until day 330 (weaning; 236 ± 19 days of age). Effects of treatment × day tended (*P *= 0.08) to be detected for supplement DM intake. *P ≤ 0.03.

Effects of day and block, but not treatment × day and treatment (*P *≥ 0.29), were detected (*P *≤ 0.03) for herbage mass, herbage allowance, and forage in vitro digestible organic matter and crude protein (Table 2). Pastures in block 1 had greater (*P *≤ 0.03) average herbage mass (6449 ± 138 vs. 4546 ± 202 kg DM/ha, respectively) and herbage allowance (6.77 ± 0.34 vs. 5.10 ± 0.49 kg DM/kg BW, respectively) compared to pastures in block 2. Pastures in block 1 had greater average concentrations of in vitro digestible organic matter (39.5 ± 0.64 vs. 35.1 ± 0.45%, respectively) and crude protein (10.7 ± 0.27 vs. 9.0 ± 0.20%, respectively) compared to pastures in block 2. Effects of treatment × day tended (*P *= 0.08) to be detected for supplement DM intake of cows and calves (Fig. 1). supplement DM intake was greater (*P *≤ 0.03) for BAC vs. CON cow-calf pairs on days 42 to 48, 56 to 62, 168 to 174, and 266 to 272, and did not differ (*P *≥ 0.14) between treatments during all remaining days of the study (Fig. 1). Average supplement DM intake did not differ (*P *= 0.11) between treatments (52 ± 1.7 vs. 56 ± 1.7 g/day for CON and BAC, respectively).

**Table 2.** Herbage mass and allowance, forage in vitro organic matter digestibility and crude protein of bahiagrass pastures.^a^

Item	Treatment^b^			*P*-value		
	CON	BAC	SEM	Treatment × day	Treatment	Day
**Herbage mass, kg DM/ha**						
** Day 0**	6057	6177	223	0.72	0.67	<0.01
** Day 30**	6865	6774	223	…	…	…
** Day 60**	6585	6509	223	…	…	…
** Day 90**	4183	3789	223	…	…	…
** Day 210**	5165	5416	223	…	…	…
** Day 240**	6057	5705	223	…	…	…
** Day 270**	4591	4293	223	…	…	…
** Day 300**	4892	4897	223	…	…	…
**Herbage allowance, kg DM/kg BW**						
** Day 0**	7.62	7.71	0.39	0.60	0.86	<0.01
** Day 30**	7.96	7.91	0.39	…	…	…
** Day 60**	7.70	7.61	0.39	…	…	…
** Day 90**	4.83	4.55	0.39	…	…	…
** Day 210**	5.33	5.71	0.39	…	…	…
** Day 240**	5.88	5.39	0.39	…	…	…
** Day 270**	4.22	3.89	0.39	…	…	…
** Day 300**	4.35	4.29	0.39	…	…	…
**In vitro digestible organic matter, %**						
** Day 0**	36.0	35.2	1.34	0.81	0.88	<0.01
** Day 30**	31.7	31.7	1.34	…	…	…
** Day 60**	27.1	26.5	1.34	…	…	…
** Day 90**	28.2	28.1	1.34	…	…	…
** Day 210**	38.6	39.9	1.34	…	…	…
** Day 240**	39.2	36.2	1.34	…	…	…
** Day 270**	47.6	49.9	1.34	…	…	…
** Day 300**	51.3	50.4	1.34	…	…	…
**Crude protein, % of DM**						
** Day 0**	9.87	10.03	0.58	0.97	0.64	<0.01
** Day 30**	7.67	7.76	0.58	…	…	…
** Day 60**	5.81	5.69	0.58	…	…	…
** Day 90**	4.66	5.15	0.58	…	…	…
** Day 210**	11.2	11.9	0.58	…	…	…
** Day 240**	9.92	9.55	0.58	…	…	…
** Day 270**	14.2	15.1	0.58	…	…	…
** Day 300**	14.5	14.6	0.58	…	…	…

a Herbage mass and hand-plucked samples of pastures were collected every 30 days from day 0 to 300, except when herbage mass was limited between day 90 and 210.

b Treatments consisted of cow-calf pairs offered free-choice access to a trace mineral supplement, either alone (**CON**) or combined (**BAC**) with a *Bacillus*-based DFM supplement (3 g/head/day) from day 0 (94 ± 19 days prepartum) until day 330 (weaning; 236 ± 19 days of age).

### Maternal performance

Effects of treatment × day were detected (*P *= 0.05) for cow BCS (Table 3). Cow BCS on day 240 was greater (*P *= 0.01) for BAC vs. CON cows and did not differ (*P *≥ 0.48) between treatments during all remaining days (Table 3). Effects of treatment × day and treatment were not detected (*P *≥ 0.55) for cow BW (Table 3). Cow BW change did not differ (*P *≥ 0.16) between treatments (Table 3). Cow BCS change from day 0 to 60 and day 60 to 150 did not differ (*P *≥ 0.29) between treatments (Table 3). Cow BCS change from day 150 to 240 was greater (*P *= 0.01) for BAC vs. CON cows, whereas cow BCS change from day 240 to 330 was greater (*P *< 0.01) for CON vs. BAC cows (Table 3). Effects of treatment were not detected (*P *≥ 0.13) for percentage of cows pregnant with a second offspring, calving percentage, calving date, and percentage of male calves in the first and second offspring (Table 4). Effects of treatment × day and treatment were not detected (*P *≥ 0.38) for plasma concentrations of cortisol, glucose, urea N, NEFA, and IGF-1 in cows (Table 5).

**Table 3.** Body weight (**BW**) and body condition score (**BCS**) of cows offered free-choice access to a trace mineral supplement, either alone (**CON**) or combined (**BAC**) with a *bacillus*-based DFM supplement (3 g/head/day) from day 0 (94 ± 19 days prepartum) until day 330 (weaning; 236 ± 19 days of age).

Item	Treatment				*P*-value		
	CON	BAC	SEM	*P* ^1^	Treatment × day	Treatment	Day
**Cow BW, kg**							
** Day 0**	526	526	4.0	0.99	0.55	0.76	<0.01
** Day 60 (near calving)**	576	575	4.0	0.90	…	…	…
** Day 150 (start of the breeding season)**	498	501	4.0	0.59	…	…	…
** Day 240 (end of the breeding season)**	512	511	4.0	0.84	…	…	…
** Day 330 (weaning)**	537	529	4.0	0.18	…	…	…
**Cow BCS (scale 1 to 9)**							
** Day 0**	5.32	5.37	0.08	0.67	0.05	0.43	<0.01
** Day 60 (near calving)**	5.68	5.76	0.08	0.55	…	…	…
** Day 150 (start of the breeding season)**	4.87	4.86	0.08	0.91	…	…	…
** Day 240 (end of the breeding season)**	4.83	5.13	0.08	0.01	…	…	…
** Day 330 (weaning)**	5.29	5.20	0.08	0.48	…	…	…
**Cow BW change, kg**							
** Day 0 to 60**	50	51	2.4	…	…	0.79	…
** Day 60 to 150**	−78	−74	4.2	…	…	0.52	…
** Day 150 to 240**	9	13	2.9	…	…	0.35	…
** Day 240 to 330**	24	19	3.0	…	…	0.16	…
**Cow BCS change**				…	…		…
** Day 0 to 60**	0.39	0.39	0.08	…	…	0.99	…
** Day 60 to 150**	−0.77	−0.90	0.08	…	…	0.29	…
** Day 150 to 240**	−0.04	0.27	0.08	…	…	0.01	…
** Day 240 to 330**	0.45	0.06	0.08	…	…	<0.01	…

a *P*-value for the comparison of treatments within each respective day of the study for cow BW and BCS.

**Table 4.** Reproductive performance of cows offered free-choice access to a trace mineral supplement, either alone (**CON**) or combined (**BAC**) with a *bacillus*-based DFM supplement (3 g/head/day) from day 0 (94 ± 19 days prepartum) until day 330 (weaning; 236 ± 19 days of age).

Item	Treatment			*P*-value
	CON	BAC	SEM	Treatment
**First offspring^a^**				
** Calving,^b^ % of total**	100.0	99.0	0.67	0.29
** Calving date, day of the study**	93	95	1.6	0.32
** Male calves, % of total**	42.6	49.8	4.4	0.26
**Second offspring^a^**				
** Pregnant on day 280, % of total**	94.6	91.1	2.13	0.25
** Calving,^b^ % of total**	94.5	89.9	2.22	0.13
** Calving date, day of the study**	454	454	2.8	0.98
** Male calves, % of total**	53.9	53.1	5.0	0.91
** Birth BW, kg**	36.3	36.4	0.48	0.81

a First offspring of the study comprised of calves that were in utero from day 0 to approximately day 94. Second offspring of the study comprised of calves that were conceived during the breeding season (day 150 to 240).

b Calving percentage consists of cows that were diagnosed as pregnant and delivered a live calf at birth. Cows that did not deliver a live calf at birth were removed from the statistical analyses of all post-calving data of cows.

**Table 5.** Blood data of cows and their calves offered free-choice access to a trace mineral supplement, either alone (**CON**) or combined (**BAC**) with a *bacillus*-based DFM supplement (3 g/head/day) from day 0 (94 ± 19 days prepartum) until day 330 (weaning; 236 ± 19 days of age).

Item	Treatment				*P*-value		
	CON	BAC	SEM	*P* ^a^	Treatment × day	Treatment	Day
**Cow plasma cortisol, ng/mL**							
** Day 0**	18.9	18.5	1.8	0.88	0.83	0.66	<0.01
** Day 30**	25.9	27.2	1.8	0.59	…	…	…
** Day 60**	10.1	11.3	1.8	0.61	…	…	…
**Cow plasma glucose, mg/dL**							
** Day 0**	81.8	81.1	1.21	0.71	0.40	0.38	<0.01
** Day 30**	81.8	82.3	1.21	0.79	…	…	…
** Day 60**	81.3	78.7	1.21	0.13	…	…	…
**Cow plasma urea N, mg/dL**							
** Day 0**	11.6	11.4	0.71	0.87	0.53	0.49	<0.01
** Day 30**	12.9	13.9	0.71	0.33	…	…	…
** Day 60**	8.71	9.55	0.71	0.43	…	…	…
**Cow plasma NEFA, mEq/L**							
** Day 0**	0.339	0.345	0.028	0.88	0.64	0.98	<0.01
** Day 30**	0.344	0.359	0.028	0.72	…	…	…
** Day 60**	0.457	0.435	0.028	0.59	…	…	…
**Cow plasma IGF-1, ng/mL**							
** Day 0**	28.8	28.6	0.89	0.88	0.67	0.70	<0.01
** Day 30**	29.9	29.9	0.89	0.97	…	…	…
** Day 60**	30.8	31.9	0.89	0.36	…	…	…
**Calf blood data at birth^b^**							
** Plasma cortisol, mg/mL**	12.7	16.9	2.40	…	…	0.22	…
** Plasma haptoglobin, mg/mL**	0.095	0.104	0.019	…	…	0.75	…
** Serum IgG, mg/mL**	42.2	48.3	2.7	…	…	0.05	…

a *P*-value for the comparison of treatments within each respective day of the study for cow plasma data.

b Blood samples from jugular vein were collected from three female calves per pasture within the first 24 hours of birth but after colostrum consumption.

### Offspring preweaning performance

Effects of treatment were not detected (*P *≥ 0.22) for calf plasma concentrations of haptoglobin and cortisol at birth (Table 5). Serum concentrations of IgG at birth were greater (*P *= 0.05) for BAC vs. CON calves (Table 5). Calf preweaning BW and ADG were covariate-adjusted for effects of calf sex and age (*P *≤ 0.03). Effects of treatment × day were detected (*P *= 0.05) for calf preweaning BW, which was greater (*P *= 0.03) for BAC vs. CON calves on day 330 and did not differ (*P *≥ 0.21) between treatments at birth and on days 150 and 240 (Table 6). Calf preweaning ADG from birth to day 150 and from day 240 to 330 did not differ (*P *≥ 0.42) between treatments. Calf preweaning ADG from day 150 to 240 and from birth to day 330 were greater (*P *≤ 0.05) for BAC vs. CON calves (Table 6).

**Table 6.** Preweaning growth performance of calves from cow-calf pairs that were offered free-choice access to a trace mineral supplement, either alone (**CON**) or combined (**BAC**) with a *bacillus*-based DFM supplement (3 g/head/day) from day 0 (94 ± 19 days prepartum) until day 330 (weaning; 236 ± 19 days of age).

Item	Treatment				*P*-value		
	CON	BAC	SEM	*P* ^a^	Treatment × day	Treatment	day
**First offspring BW,^b^ kg**							
** Birth**	36	36	1.7	0.84	0.02	0.84	<0.01
** Day 150 (start of the breeding season)**	87	88	1.7	0.99	…	…	…
** Day 240 (end of the breeding season)**	155	158	1.7	0.21	…	…	…
** Day 330 (weaning)**	252	257	1.7	0.03	…	…	…
**First offspring ADG,^b^ kg/day**							
** Birth to day 150**	0.92	0.93	0.01	…	…	0.95	…
** Day 150 to 240**	0.79	0.83	0.01	…	…	0.02	…
** Day 240 to 335**	1.06	1.08	0.01	…	…	0.42	…
** Birth to day 330**	0.91	0.94	0.01	…	…	0.05	…

a *P*-value for the comparison of treatments within each respective day of the study for calf BW.

b Calf BW and ADG were covariate-adjusted for effects of calf sex and age (*P *≤ 0.03).

### Steer post-weaning preconditioning

Effects of treatment × day and treatment were not detected (*P *≥ 0.18) for steer BW, concentrate DM intake, hay DM intake, and total DM intake (Table 7). Steer ADG and gain: feed did not differ (*P *≥ 0.15) between treatments (Table 7). Effects of treatment × day and treatment were not detected (*P *≥ 0.12) for plasma concentrations of haptoglobin and IGF-1, serum titers and seroconversion against BVDV-1 and BVDV-2, and seroconversion against IBR and PI-3 (Table 8). Effects of treatment × day were detected (*P *≥ 0.03) for plasma concentrations of cortisol, which was greater (*P *= 0.01) for CON vs. BAC steers on day 398 and did not differ (*P *≥ 0.23) between treatments on all remaining days (Table 8). Effects of treatment × day tended (*P *= 0.10) to be detected for serum titers against IBR and PI-3, which both were greater (*P *= 0.05) for BAC vs. CON steers on day 369 and did not differ (*P *≥ 0.21) between treatments on days 350 and 398 (Table 8).

**Table 7.** Growth performance during preconditioning (day 350 to 398) and feedlot periods (day 399 to 609) of steers selected from cow-calf pairs that were offered free-choice access to a trace mineral supplement, either alone (**CON**) or combined (**BAC**) with a *bacillus*-based DFM supplement (3 g/head/day) from day 0 (94 ± 19 days prepartum) until day 330 (weaning; 236 ± 19 days of age).

Item^a^	Treatment			*P*-value		
	CON	BAC	SEM	Treatment × day	Treatment	Day
**Preconditioning (day 350 to 398)**						
**Steer BW,^b^ kg**						
** Day 350 (start of preconditioning)**	266	260	7.2	0.56	0.33	<0.01
** Day 398 (end of preconditioning)**	334	322	7.2	…	…	…
**ADG,^b^ kg/day**	1.33	1.23	0.05	…	0.15	…
**Concentrate DMI, kg/day**	4.22	4.14	0.12	0.39	0.61	<0.01
**Hay DMI, kg/day**	2.27	2.10	0.08	0.84	0.18	<0.01
**Total DMI, kg/day**	6.49	6.22	0.18	0.69	0.33	<0.01
**Gain: feed**	0.209	0.206	0.009	…	0.82	…
**Feedlot (day 399 to 609)**	…	…	…	…	…	…
**Steer BW,^b^ kg**						
** Day 399 (feedlot arrival)**	301	296	8.8	0.61	0.91	<0.01
** Day 407 (start of growing period)**	304	301	8.8	…	…	…
** Day 382 (end of growing period)**	426	417	8.8	…	…	…
** Day 609 (end of finishing period)**	570	566	8.8	…	…	…
**Post-transportation BW shrink (%)^c^**	8.87	8.48	0.29	…	0.35	…
**ADG,^b^ kg/day**						
** Growing period**	1.61	1.53	0.06	…	0.33	…
** Finishing period**	1.15	1.19	0.05	…	0.57	…
** Overall**	1.29	1.28	0.04	…	0.91	…
**DMI, kg/day**						
** Growing period**	8.12	7.75	0.22	…	0.26	…
** Finishing period**	7.84	8.08	0.36	…	0.65	…
** Overall**	7.94	7.95	0.29	…	0.98	…
**Gain: feed**						
** Growing period**	0.199	0.196	0.005	…	0.70	…
** Finishing period**	0.150	0.149	0.009	…	0.96	…
** Overall**	0.168	0.167	0.007	…	0.86	…

a From day 330 to 349, steers were provided concentrate DM supplementation at 0.5%, 1.0%, and 1.5% of BW for 7, 7, and 6 days, respectively. On day 350, steers (*n *= 2 to 3 steers per preweaning pasture) were assigned to a preconditioning period in drylot (day 350 to 398) and provided free choice access to limpograss hay and concentrate DM supplementation at 1.5% of BW. On day 398, steers were transported for 1193 km over 14 hours to the feedlot facility. Immediately after arrival (day 399), steers were weighed, assigned to feedlot pens according to preweaning pasture group, and provided free choice access to corn silage during an 8-day adaptation period (day 399 to 406). Steers were then fed the growing diet for 76 days (day 407 to 482), followed by the finishing diet for 126 days (day 483 to 609). On day 609, steers were transported for 790 km over 9 hours to the packing facility. Steers were slaughtered on day 610.

b Covariate-adjusted for age at weaning (*P *≤ 0.05).

c Body weight shrink was calculated on day 399 relative to BW on day 398, following the 14-hour transportation period.

**Table 8.** Plasma and serum data during preconditioning (day 350 to 398) of steers selected from cow-calf pairs that were offered free-choice access to a trace mineral supplement, either alone (**CON**) or combined (**BAC**) with a *bacillus*-based DFM supplement (3 g/head/day) from day 0 (94 ± 19 days prepartum) until day 330 (weaning; 236 ± 19 days of age).

Item^a^	Treatment				*P*-value		
	CON	BAC	SEM	*P* ^b^	Treatment × day	Treatment	Day
**Plasma IGF-1, ng/mL**							
** Day 350**	139	126	12.2	0.47	0.65	0.19	<0.01
** Day 352**	104	91	12.2	0.45	…	…	…
** Day 355**	155	145	12.2	0.50	…	…	…
** Day 358**	130	114	12.2	0.16	…	…	…
** Day 369**	103	108	12.2	0.60	…	…	…
** Day 398**	113	117	12.2	0.66	…	…	…
**Plasma cortisol, ng/mL**							
** Day 350**	14.4	13.1	2.07	0.65	0.03	0.39	<0.01
** Day 352**	20.6	17.1	2.07	0.23	…	…	…
** Day 355**	16.6	17.8	2.07	0.67	…	…	…
** Day 358**	18.1	19.2	2.07	0.70	…	…	…
** Day 369**	17.4	15.6	2.07	0.54	…	…	…
** Day 398**	29.2	21.7	2.07	0.01	…	…	…
**Plasma haptoglobin, mg/dL**							
** Day 350**	0.19	0.13	0.049	0.39	0.92	0.55	<0.01
** Day 352**	1.21	1.20	0.049	0.86	…	…	…
** Day 355**	0.89	0.90	0.049	0.93	…	…	…
** Day 358**	0.49	0.41	0.049	0.31	…	…	…
** Day 369**	0.08	0.08	0.049	0.94	…	…	…
** Day 398**	0.04	0.05	0.049	0.90	…	…	…
**BVDV-1 serum titers, log_2_**							
** Day 350**	1.65	1.98	0.395	0.56	0.68	0.79	<0.01
** Day 369**	1.38	1.62	0.395	0.67	…	…	…
** Day 398**	5.43	5.12	0.395	0.59	…	…	…
**BVDV-1 seroconversion, % of total**							
** Day 350**	23.5	26.5	7.72	0.79	0.87	0.54	<0.01
** Day 369**	18.9	26.5	7.72	0.49	…	…	…
** Day 398**	91.7	90.1	7.72	0.88	…	…	…
**BVDV-2 serum titers, log_2_**							
** Day 350**	2.14	2.09	0.302	0.92	0.79	0.49	<0.01
** Day 369**	1.18	1.36	0.302	0.67	…	…	…
** Day 398**	7.32	7.68	0.302	0.39	…	…	…
**BVDV-2 seroconversion, % of total**							
** Day 350**	63.6	45.5	7.94	0.11	0.12	0.83	<0.01
** Day 369**	13.6	27.3	7.94	0.23	…	…	…
** Day 398**	100.0	100.0	7.94	0.99	…	…	…
**IBR serum titers, log_2_**					…	…	…
** Day 350**	1.09	1.00	0.205	0.75	0.10	0.17	<0.01
** Day 369**	1.27	1.82	0.205	0.05	…	…	…
** Day 398**	1.86	2.23	0.205	0.21	…	…	…
**IBR seroconversion, % of total**							
** Day 350**	9.10	0	8.89	0.47	0.63	0.99	<0.01
** Day 369**	27.3	31.8	8.89	0.72	…	…	…
** Day 398**	45.5	50.0	8.89	0.72	…	…	…
**PI-3 serum titers, log_2_**							
** Day 350**	4.45	5.09	0.448	0.32	0.10	0.26	<0.01
** Day 369**	4.82	6.00	0.448	0.05	…	…	…
** Day 398**	9.82	9.41	0.448	0.52	…	…	…
**PI-3 seroconversion, % of total**							
** Day 350**	95.5	90.9	3.16	0.31	0.71	0.56	0.05
** Day 369**	100.0	100.0	3.16	0.99	…	…	…
** Day 398**	100.0	100.0	3.16	0.99	…	…	…

a On day 350, 24 steers per treatment were randomly allocated into 1 of 16 drylot pens (3 steers/pen), using the same previous preweaning pasture distribution. On day 350, all steers were treated with doramectin for internal and external parasites (Dectomax, Zoetis Inc.) and vaccinated against pathogens associated with bovine respiratory disease (Bovi Shield One Shot, Zoetis Inc.), and clostridium (Ultrabac 7, Zoetis Inc.). On day 369, steers received booster vaccinations of Bovi Shield Gold 5 and Ultrabac 7 (Zoetis Inc.).

b *P*-value for the comparison of treatments within each respective day of the study.

### Steer post-weaning feedlot

Effects of treatment × day and treatment were not detected (*P *≥ 0.61) for steer BW during the feedlot period (Table 7). Effects of treatment were not detected (*P *≥ 0.26) for post-transportation BW shrink, steer ADG, DM intake, and gain: feed during the feedlot period (Table 7). Effects of treatment were not detected (*P *≥ 0.16) for all carcass characteristics, except for percentage of carcasses grading select, low choice, and prime (Table 9). The percentage of carcasses grading Select was less (*P *= 0.05), whereas the percentage of carcasses grading Low Choice and Prime were greater (*P *≤ 0.05) for BAC vs. CON steers (Table 9). The percentage of carcasses grading Low Choice or above was greater for BAC vs. CON steers (Table 9).

**Table 9.** Carcass characteristics of steers selected from cow-calf pairs that were offered free-choice access to trace mineral supplement, either alone (**CON**) or combined (**BAC**) with a *bacillus*-based DFM supplement (3 g/head/day) from day 0 (94 ± 19 days prepartum) until day 330 (weaning; 236 ± 19 days of age).

Item	Treatment			*P*-value
	CON	BAC	SEM	Treatment
**Hot carcass weight, kg**	339	334	6.7	0.62
**Dressing percent,^a^ %**	59.0	58.9	0.29	0.86
**12^th^ rib fat thickness, cm**	0.87	0.89	0.023	0.55
**Longissimus muscle area, cm^b^**	79.3	81.3	1.48	0.35
**Kidney, pelvic, and heart fat, %**	2.47	2.21	0.129	0.16
**Yield grade**	3.40	3.33	0.136	0.70
**Marbling^b^**	616	642	23.6	0.43
**Prime**	0	13.6	5.42	0.05
**High choice, %**	23.3	9.33	9.29	0.31
**Average choice, %**	43.7	35.6	12.4	0.65
**Low choice, %**	9.2	36.7	8.74	0.03
**Select, %**	24.0	4.40	7.33	0.05
**Low choice or above, %**	76.0	95.6	7.33	0.05

a Dressing percent calculated from dividing the average final BW on days 608 and 609 (before transportation to packing facility for harvest) by the hot carcass weight.

b 500 = small; 600 = modest; 700 = moderate (Engle et al 2000).

### Heifer post-weaning performance

**Fig. 2.**
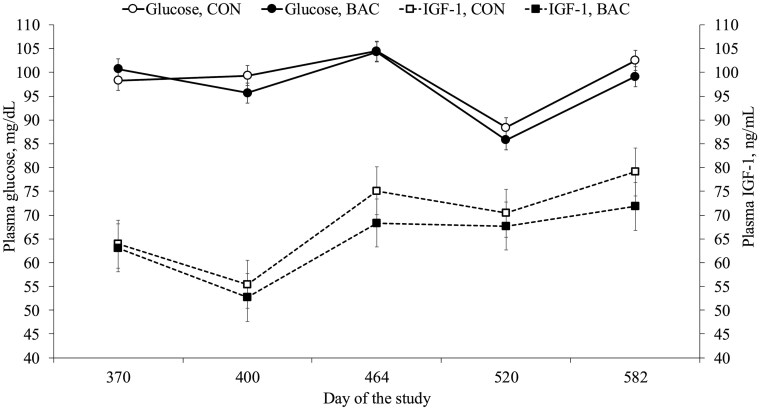
Post-weaning plasma concentrations of glucose and IGF-1 of heifers born from cows offered free-choice access to trace mineral supplement, either alone (**CON**) or combined (**BAC**) with a DFM supplement (3 g/head/day) containing a mixture of *Bacillus subtilis* and *B. licheniformis* (6.6 × 10^9^ CFU per day) from day 0 (94 ± 19 days prepartum) until day 330 (weaning; 236 ± 19 days of age). Concentrate DM supplementation was offered at 1.5% of BW from day 350 to 615. Heifers were assigned to an estrus synchronization protocol starting on day 464. Heifers detected in estrus were inseminated (AI) 12 hours after estrus detection. On day 478, heifers not detected in estrus were fixed-time AI. Bulls were placed with heifers from day 485 to 582. Effects of treatment × day and treatment were not detected (*P *≥ 0.29) for plasma concentrations of glucose and IGF-1.

Effects of treatment × day and treatment were not detected (*P *≥ 0.26) for heifer BW, reproductive tract score, puberty attainment (Table 10), and plasma concentrations of glucose and IGF-1 (Fig. 2). Heifer pre-breeding ADG was greater (*P *= 0.03) for CON vs. BAC heifers, whereas the percentage of mature BW at breeding, ADG during the breeding season, and overall ADG did not differ (*P *≥ 0.17) between treatments (Table 10). Percentage of heifers detected in estrus was greater (*P *= 0.05) for CON vs. BAC heifers, but the pregnancy percentages (AI and final) did not differ (*P *≥ 0.60) between treatments (Table 10).

**Table 10.** Post-weaning growth and reproduction of heifers selected from cow-calf pairs that were offered free-choice access to a trace mineral supplement, either alone (**CON**) or combined (**BAC**) with a *bacillus*-based DFM supplement (3 g/head/day) from day 0 (94 ± 19 days prepartum) until day 330 (weaning; 236 ± 19 days of age).

Item^a^	Treatment			*P*-value		
	CON	BAC	SEM	Treatment × day	Treatment	Day
**Heifer BW,^b^ kg**						
** Day 350**	256	259	5.9	0.26	0.54	<0.01
** Day 434**	330	327	5.9	…	…	…
** Day 464 (start of the estrus synchronization)**	345	338	5.9	…	…	…
** Day 475 (start of the breeding season)**	388	378	5.9	…	…	…
** Day 520 (AI pregnancy diagnosis)**	430	419	5.9	…	…	…
** Day 582 (end of the breeding season)**	419	409	5.9	…	…	…
** Day 615 (final pregnancy diagnosis)**	459	450	5.9	…	…	…
**ADG, kg/day**						
** Pre-breeding (day 350 to 475)^b^**	0.71	0.65	0.020	…	0.03	…
** During breeding (day 475 to 582)**	0.80	0.79	0.021	…	0.66	…
** Overall (day 350 to 615)**	0.76	0.73	0.018	…	0.17	…
**% of mature BW at breeding^c^**	68.9	67.6	1.16	…	0.40	…
**Reproductive tract score, scale 1 to 5**						
** Day 434**	3.31	3.20	0.200	0.87	0.68	<0.01
** Day 464 (start of the estrus synchronization)**	4.00	3.94	0.200	…	…	…
**Pubertal, % of total heifers^d^**						
** Day 434**	21.9	21.7	7.95	0.39	0.46	0.03
** Day 464 (start of the estrus synchronization)**	43.8	30.8	7.95	…	…	…
**Detected in estrus (day 475 to 478)**	68.8	43.3	9.47	…	0.05	…
**Pregnant, % of total heifers**						
** AI**	45.6	48.2	9.89	…	0.85	…
** Final (AI + bull)**	86.7	81.8	5.67	…	0.60	…

a On day 350, 32 heifers per treatment were stratified by previous pre-weaning pasture distribution and allocated into 1 of 16 bahiagrass pastures (1 ha and 4 heifers per pasture). Estrus activity was detected from days 475 to 478. Heifers detected in estrus were inseminated (AI) 12 hours after estrus detection. On day 478, all heifers previously not observed in estrus were fixed-time AI. Yearling Angus bulls were placed with heifers from day 485 to 582 (1 bull per pasture).

b Covariate-adjusted (*P *≤ 0.04) for heifer age at weaning.

c Assuming a mature BW of 499 kg (Moriel et al 2024).

d Percentage of heifers with a detectable corpus luteum via transrectal ultrasonography.

## Discussion

### Maternal performance

The present study builds on Izquierdo et al. (2024), who showed that exclusive maternal consumption of *Bacillus*-based DFM from 139 days prepartum to 104 days postpartum improved cow and calf performance. In the present study, we extended the supplementation period to a full year, beginning in the third trimester and continuing through postpartum and lactation, and shifted delivery from hand-fed by-product to free-choice trace mineral supplementation available to both cows and calves. These changes aimed to improve the duration, reach, and practical applications of DFM supplementation in extensive cow-calf systems.

Supplement DM intake per cow-calf pair varied throughout the study, reaching levels below the expected intake from day 119 to 272, and with BAC pastures showing greater supplement DM intake in four weeks of the study. In the present study, the reduced intake of trace mineral supplements was likely influenced by season (lower during fall/winter vs. summer; Arthington and Ranches 2021) and the concurrent provision of sugarcane molasses + urea supplement (McDowell 1996; Ranches et al 2021). Although the exact individual supplement DM intake by cows and their calves cannot be determined, average trace mineral supplement DM intake met the expected level and did not differ between treatments. These findings suggest that BAC cows consumed the expected *Bacillus*-based DFM intake for most of the study.

Unlike Izquierdo et al. (2024), BAC did not improve prepartum BCS gain and BCS at calving. This discrepancy may be associated with the differences in animal category (multiparous cows in this study vs. nulliparous heifers in Izquierdo et al 2024), given that dam parity affects maternal metabolic demands (NASEM 2016), with younger cows showing reduced nutrient partitioning to fetal growth (Duncan et al 2023). During the breeding season, CON cows lost BCS while BAC cows gained BCS. However, CON cows regained BCS post-breeding, leading to similar BCS at weaning between treatments. The BAC supplementation was not provided during the breeding season in Izquierdo et al. (2024), preventing direct comparison. However, the breeding season in the present study coincided with winter, when cows received low-quality hay. Although in vitro results should be interpreted cautiously, previous studies using the same *Bacillus*-based DFM showed variable effects on DM and fiber digestibility depending on forage in vitro digestibility and crude protein (Pan et al 2022; Cappellozza et al 2023b). The inconsistent effects on BCS change of multiparous cows suggest that the benefits of BAC supplementation on forage digestibility are perhaps more apparent under nutritionally challenging conditions. Despite BCS improvements during breeding, pregnancy rates did not differ between treatments, agreeing with Izquierdo et al. (2024). This may indicate that modest BCS gain during the breeding season was insufficient to enhance reproductive performance in BAC cows already calving in good condition. Similarly, energy and protein supplementation for beef cows in the same research site increased pregnancy only in cows with BCS < 5, but not in those with BCS ≥ 5 (Moriel et al 2024).

Maternal protein and energy supplementation during late gestation can alter maternal plasma indicators of energy and protein metabolism (Palmer et al 2020, 2022). The detailed plasma metabolome data from cows and their calves during pre- and post-partum periods will be presented in a follow-up manuscript. However, BAC supplementation did not affect any plasma variables of cows measured herein during the prepartum period. In contrast, previous studies reported improved metabolic efficiency in BAC-supplemented beef heifers, including greater plasma concentrations of glucose during prepartum and early post-partum periods (Izquierdo et al 2024) and increased prepartum concentrations of amino acids including methionine, glycine, and phenylalanine (Izquierdo et al 2025). Differences in parity and forage conditions, described above, likely contributed to the divergent maternal plasma responses observed here and in prior studies.

### Offspring pre- and post-weaning performance

In the present study, plasma cortisol and haptoglobin concentrations at birth did not differ between CON and BAC calves, reflecting similar maternal plasma concentrations of cortisol during the prepartum period and suggesting comparable prenatal stress. Calf birth BW was also unaffected by BAC supplementation, consistent with Izquierdo et al. (2024). Although no treatment effects were observed for maternal BCS, BAC calves had greater serum IgG concentrations within 24 hours of birth compared with CON calves, suggesting improved passive immunity. This contrasts with Izquierdo et al. (2024), who reported no effect of maternal BAC supplementation on calf serum IgG. Nonetheless, BAC supplementation to dairy cows for 30 days precalving did not change the volume of colostrum produced but improved the quality of the colostrum and enhanced calf serum IgG concentrations for up to 14 days following birth (Silva et al 2025). The mechanisms underlying the increased serum IgG concentrations in BAC vs. CON calves remain unclear.

Contrary to Izquierdo et al. (2024), where maternal BAC supplementation had no effect on calf preweaning growth, the present study observed greater BW at weaning for BAC vs. CON calves. In both studies, calf ADG from birth to 3 months of age (start of the breeding season) did not differ between treatments, suggesting similar dam milk production and composition during this period. The enhanced preweaning growth observed here likely results from the direct calf consumption of BAC in addition to differences in dam parity and supplementation duration. In Izquierdo et al. (2024), nulliparous heifers were supplemented from mid-gestation through early postpartum, but calves were early weaned at 3 months and did not consume BAC directly. In contrast, calves in the current study were nursing and had free-choice access to BAC supplements until weaning. Although BW at the start of the breeding season was similar, BAC calves showed greater ADG during this period and heavier BW at weaning. These results were likely influenced by the improved maternal BCS and potentially enhanced milk production during the breeding season of BAC cows, combined with the additive effects of direct supplement intake by BAC calves. Together, these findings suggest that both maternal and early-life exposure to *Bacillus*-based DFM can improve calf postnatal growth performance. Moreover, direct consumption of *Bacillus*-based DFM during the preweaning period may be critical to reveal growth-promoting effects under forage-based systems.

Growth performance during the preconditioning period did not differ between BAC and CON steers, likely due to the moderate-concentrate diet (1.5% BW). This contrasts with Izquierdo et al. (2024), where post-weaning growth benefits were observed under higher-concentrate feeding (3.1% of BW), suggesting that post-weaning nutrition influences the expression of maternal or early-life *Bacillus*-based DFM effects. Despite differences in supplementation protocols and timing, both studies observed no treatment effects on plasma concentrations of cortisol or haptoglobin, except for a minor reduction in plasma cortisol in BAC calves at the end of preconditioning in the current study. This is consistent with previous findings in *Bos indicus*-influenced cattle, where prenatal nutritional interventions had minor effects on hypothalamic–pituitary–adrenal axis responsiveness (Moriel et al 2020; Palmer et al 2020, 2022; Izquierdo et al 2022, 2023; Vedovatto et al 2022). Nonetheless, BAC supplementation influenced the humoral immune responses to vaccination in both studies. In the present study, BAC calves exhibited greater serum titers against IBR and PI-3 viruses 19 days after first vaccination, supporting a virus-specific programming of the humoral immune system via early-life DFM exposure. The perinatal period represents a critical window for developmental programming of physiological systems, including the immune system, through the microbiota–gut–brain axis, where early nutrition shapes microbial colonization with lasting effects on offspring health (Ratsika et al 2021). Therefore, the observed effects of previous BAC supplementation on calf post-vaccine humoral immune response likely resulted from complex early-life interactions among microbial colonization, host development, and the environment (Cappellozza et al 2025).

Prepartum and preweaning supplementation of BAC did not affect the growth performance during the growing and finishing periods, as well as the carcass yield traits, including hot carcass weight, dressing percentage, and longissimus muscle area. However, BAC supplementation altered carcass quality grade distribution, decreasing the percentage of Select carcasses and increasing the proportion of Low Choice and Prime carcasses. Combined, the percentage of carcasses grading Low Choice or above was greater for BAC vs. CON steers. These findings suggest that early-life exposure to BAC may modestly enhance carcass quality, potentially through pre- and postnatal developmental programming mechanisms influencing adipose tissue deposition and metabolism (Du et al 2010; Harvey et al 2021; Moriel et al 2021). Additional research is warranted to confirm these effects and elucidate the underlying biological pathways responsible for the observed shifts in carcass quality grade.

Nutrition changes to maternal diet during pregnancy and preweaning periods can modulate the reproductive axis development of heifers, leading to improved growth and reproductive efficiency during their first breeding season (Moriel et al 2014; Cardoso et al 2020; Harvey et al 2021). Early-life exposure to BAC supplementation slightly reduced pre-breeding ADG but did not affect the percentage of mature BW at the start of breeding. Puberty attainment and reproductive tract scores did not differ between treatments, but early-life exposure to BAC supplementation reduced the percentage of heifers detected in estrus, possibly influenced by their lower pre-breeding growth performance (Brauner et al 2024) compared with CON heifers. Although plasma concentrations of glucose and IGF-1 did not differ between treatments, the greater pre-breeding ADG of CON heifers may have triggered a cascade of other metabolic and physiological changes positively associated with reproductive outcomes (Moriel et al 2022). Despite the lower estrus activity, pregnancy percentages were not compromised, indicating that the BAC-induced decrease in pre-breeding ADG did not translate into reduced reproductive success. These results suggest that while DFM supplementation may influence early reproductive physiology or estrus expression, it does not appear to compromise fertility outcomes in heifers achieving similar BW at the start of the breeding season.

In conclusion, extending *Bacillus*-based DFM supplementation from late gestation through the preweaning period, using free-choice trace mineral delivery, improved some aspects of cow and calf performance. Although maternal BCS, pregnancy rates, and plasma metabolic indicators were unaffected, early-life exposure to *Bacillus*-based DFM during gestation and preweaning periods enhanced the passive immunity transfer, preweaning growth, and post-vaccination humoral immune responses in calves. While no differences were observed in post-weaning growth performance or carcass yield traits of steers, BAC supplementation modestly enhanced their carcass quality grade distribution. In replacement heifers, early-life exposure to *Bacillus*-based DFM slightly reduced estrus expression without negatively impacting the percentage of pregnant heifers (AI and final). Therefore, prolonged *Bacillus*-based DFM supplementation may be a practical strategy to enhance the performance in forage-based cow-calf systems.
